# Gene-specific methylation profiles in *BRCA*-mutation positive and *BRCA*-mutation negative male breast cancers

**DOI:** 10.18632/oncotarget.24856

**Published:** 2018-04-13

**Authors:** Piera Rizzolo, Valentina Silvestri, Virginia Valentini, Veronica Zelli, Ines Zanna, Giovanna Masala, Simonetta Bianchi, Domenico Palli, Laura Ottini

**Affiliations:** ^1^ Department of Molecular Medicine, Sapienza University of Rome, Rome, Italy; ^2^ Cancer Risk Factors and Lifestyle Epidemiology Unit, Cancer Research and Prevention Institute (ISPRO), Florence, Italy; ^3^ Division of Pathological Anatomy, Department of Medical and Surgical Critical Care, University of Florence, Florence, Italy

**Keywords:** male breast cancer, promoter methylation, BRCA1/2 mutations, clinical-pathologic characteristics, pyrosequencing

## Abstract

Male breast cancer (MBC) is a rare disease. Due to its rarity, MBC research and clinical approach are mostly based upon data derived from female breast cancer (FBC). Increasing evidence indicate that on molecular level MBC may be an heterogeneous disease different from FBC.

In order to investigate whether epigenetic signatures could define molecular subgroups of MBCs, we performed promoter methylation analysis of genes involved in signal transduction and hormone signalling in *BRCA1/2* mutation-positive and -negative MBCs.

We examined 69 MBCs, paired blood samples, and 15 normal tissues for promoter methylation of *hTERT, ESR1, RASSF1, AR, MYC* and *WNT1* genes.

MBCs showed higher gene promoter methylation levels compared to paired blood and normal breast samples. Significantly higher *RASSF1* methylation levels were observed in association with *BRCA1/2* mutations, HER2 expression and high tumor grade. Significantly higher *AR* methylation levels were observed in *BRCA1/2* wild-type cases and higher *WNT1* methylation levels in PR negative cases.

Overall, our results indicate that alterations in gene methylation profiles are common in MBC and that methylation pattern of tumor-associated genes may allow for the identification of MBC molecular subgroups, that could have implications in clinical management of MBC patients.

## INTRODUCTION

Male breast cancer (MBC) is a rare disease, representing less than 1% of all breast cancers and less of 1% of all male tumors [1]. Despite the rarity, morbidity and mortality in MBC patients is a serious concern.

MBC shares some similarities with post-menopausal ER-positive female breast cancer (FBC). Increasing evidence indicate that, on clinical and molecular level, MBC may be a heterogeneous disease, different from FBC [2–4]. Compared to FBC, MBC occurs later in life, with higher stage, lower grade and more estrogen/progesterone receptor (ER/PR) positivity [5–6].

MBC research and patient management are mostly based upon data derived from its largely known female counterpart. To date, mortality and survival rates for patients with MBC have improved less over time than for patients with FBC [5]. These data highlight the need to identify specific biological markers for MBC.

Aberrant DNA methylation may play a role in the initiation of cancer, tumor progression and response to treatment [7]. Promoter methylation of genes involved in cancer development and progression, such as tumor suppressors, cell cycle regulators and transcription factors, are frequently reported aberrantly methylated in FBC [8–9].

Different studies have suggested that aberrant methylation at specific gene promoter regions may contribute to the malignant phenotype and could be used as biomarkers for diagnosis at an early stage and prediction of prognosis in breast cancer [10–12]. Moreover, there are growing evidence that the characterization of tumor-specific methylation profiles may allow for the identification of specific breast cancer subtypes. In particular, methylation analysis of FBCs allowed for the identification of gene methylation profiles associated with molecular subtypes via ER/PR, HER2 and *BRCA* mutation status [13–15].

DNA methylation abnormalities may occur in tissue adjacent to the tumor, that is considered histologically normal. A limited number of studies have showed that promoter methylation status of specific genes in normal tissue correlate with that found in tumor sample [16–17]. It is also becoming increasingly apparent that gene methylation in blood DNA of breast cancer patients may be a part of a disease predisposition mechanism [18–19]. A concordance in the promoter methylation patterns between blood DNA from breast cancer patients and corresponding tumors has been reported [20–21].

To date the contribution of aberrant DNA methylation in the pathogenesis of MBC has been investigated only in few studies [22–25]. These studies showed that methylation of genes involved in DNA repair and cell growth and differentiation may play a role in MBC and may be associated with aggressive phenotype and worse disease specific survival [22, 25].

We previously showed that *BRCA* mutation positive and *BRCA* mutation negative MBC cases display different phenotypic features, and in particular we identified a specific BRCA2-associated MBC phenotype characterized by higher tumor grade compared with MBC from the general population [26–28]. Recently, it has been reported that *BRCA2*-associated MBCs are characterized by elevated tumor methylation [25].

In this study we examined methylation profiles of *BRCA* mutation positive and *BRCA* mutation negative MBCs by performing promoter methylation analysis of a panel of breast cancer-related genes, in order to investigate whether epigenetic signatures could define molecular subgroups of MBCs.

In particular, we examined promoter methylation status of genes involved in signal transduction and hormone signalling, including *AR*, *ESR1, hTERT*, *MYC, RASSF1* and *WNT* in male breast tumors, paired blood samples and normal tissues.

Our specific aims were to examine the level of methylation of important breast cancer genes in a series of MBC cases all characterized for *BRCA1/2* mutation status and to identify potential molecular subgroups defined by their methylation patterns with clinical-pathologic correlation.

## RESULTS

### Clinical-pathologic characteristics of MBC cases

All cases included in this study were characterized for *BRCA1/2* germline mutations, the major genetic risk factor for MBC, and for the main clinical-pathologic features including: family and personal history of cancer, ER, PR, HER2 and Ki67/MIB1 expression and tumor grade (G). As shown in Table 1, 26% of MBC patients have family history of breast/ovarian cancer, 29% have personal history of cancer other than breast cancer and 14.5% are positive for *BRCA1/2* mutations. The majority of MBC cases are ER and PR positive (90.8% and 83%, respectively), HER2 and Ki67/MIB1 negative (84.2% and 61.7%, respectively) and have intermediate/moderate tumor grade (G2) (52.4%).

**Table 1:** Clinical-pathologic characteristics of the 69 male breast cancer cases analyzed

CLINICAL-PATHOLOGIC CHARACTERISTICS	N(%)
Family history of breast/ovarian cancer	
Negative	51(74.0)
Positive	18(26.0)
Tot	69
**Personal history of other cancer**	
Negative	49(71.0)
Positive	20(29.0)
Tot	69
***BRCA1/BRCA2* status**	
*BRCA1* mutated	2(2.9)
*BRCA2* mutated	8(11.6)
*BRCA1/BRCA2* wild-type	59(85.5)
Tot	69
**ER**	
Negative (≤10%)	6(9.2)
Positive (>10%)	59(90.8)
Tot	65
**PR**	
Negative (≤10%)	11(17.0)
Positive (>10%)	54(83.0)
Tot	65
**HER2**	
Negative (≤25%)	48(84.2)
Positive (>25%)	9(15.8)
Tot	57
**Ki67/MIB1**	
Negative (≤20%)	37(61.7)
Positive (>20%)	23(38.3)
Tot	60
**Hystological grade**	
G1	13(20.6)
G2	33(52.4)
G3	17(27.0)
Tot	63

### Gene promoter methylation analysis in tumor and normal tissues

Using candidate-gene approach, we examined the promoter methylation level of *AR, ESR1, hTERT*, *MYC*, *RASSF1* and *WNT1* in 69 male breast tumors and corresponding blood samples, 7 normal breast tissues and 8 normal lymph node samples.

**Figure 1:**
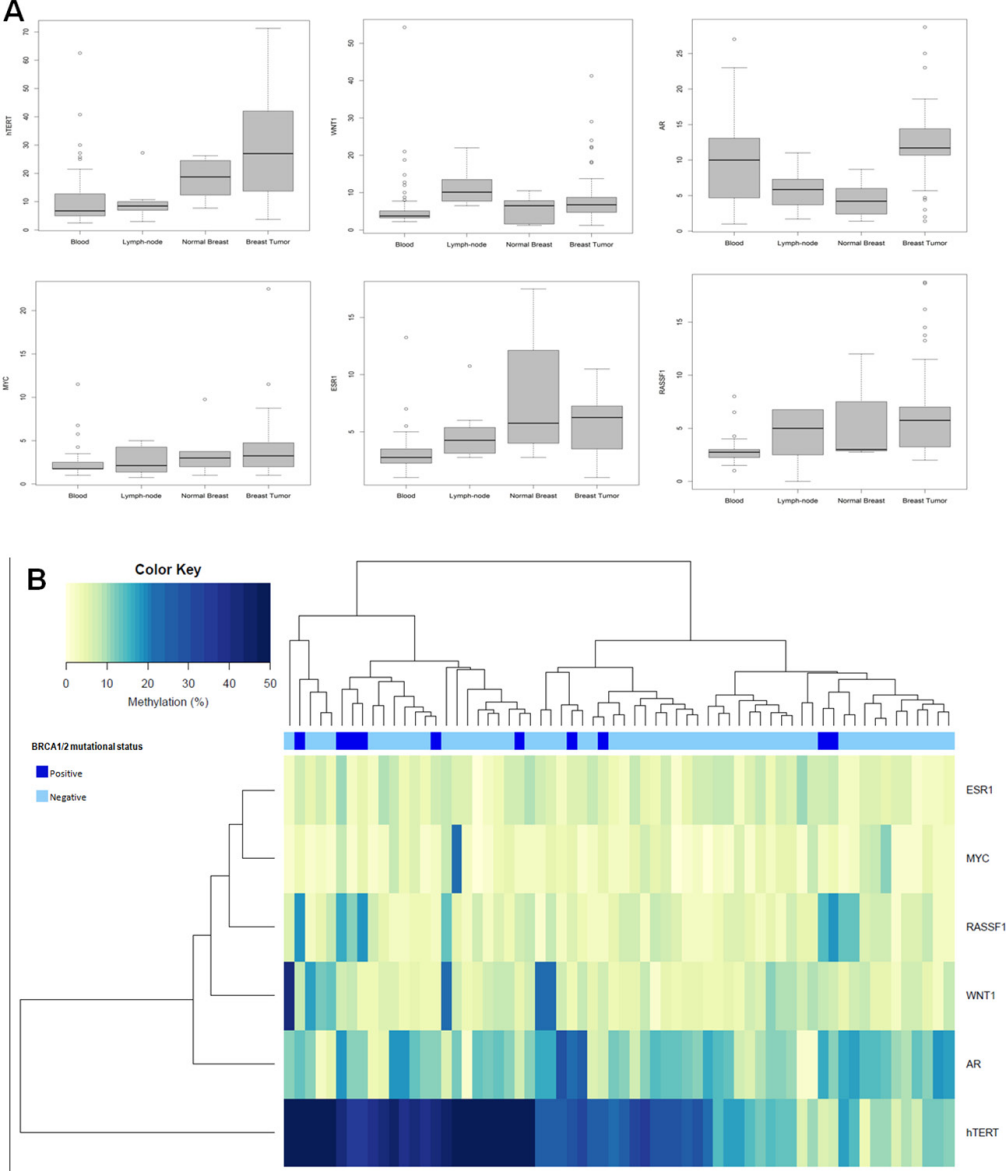
**(A)** Distribution of the methylation levels in different tissue samples from male breast cancer cases. Boxplots show comparison of median methylation levels of *hTERT*, *WNT1, AR, MYC, ESR1* and *RASSF1* genes in blood, lymph node, normal breast and tumor breast samples. **(B)** Unsupervised hierarchical clustering analysis of promoter methylation levels of 6 genes in 64 male breast turmors.

Compared with the median methylation levels of each gene in normal breast samples, 63/67 (94%) tumor samples showed higher methylation level for *AR*, 53/67 (79.1%) for *RASSF1*, 44/69 (63.8%) for *hTERT*, 37/69 (53.6%) for *MYC*, 36/68 (52.9%) for *WNT1*, and 35/68 (51.5%) for *ESR1*. Methylation levels varied among genes with predominantly high methylation levels in *hTERT*, for which levels were up to 72% in tumors and 26% in normal breast samples (Figure 1A).

Overall, tumors showed higher median methylation levels compared with normal breast tissues (Table 2). A statistically significant differences emerged for *hTERT* (p=0.0008) when we compared methylation levels of tumors with lymph node samples. *AR* median methylation levels in tumors were statistically significant higher compared to those observed in normal breast tissues and lymph node samples (p=0.0009 and p=0.003 respectively). Statistically significant differences emerged between median methylation levels of tumors and paired blood samples (Table 2).

**Table 2:** Gene-promoter median methylation levels in breast tumor, normal breast, normal lymph-node and blood samples from male breast cancer patients

	BREAST TUMOR (N=69)	NORMAL BREAST (N=7)	NORMAL LYMPH-NODE (N=8)	BLOOD (N=69)	^a^p T vs N	^a^p T vs L	^a^p T vs B
**hTERT**	27.00 (69)	18.75 (7)	8.50 (8)	6.75 (69)	0.11	**0.0008**	**<0.0001**
**AR**	11.70 (67)	4.20 (6)	5.85 (6)	10.00 (67)	**0.0009**	**0.003**	**0.0006**
**WNT1**	6.75 (68)	6.50 (7)	10.13 (8)	3.75 (68)	0.27	**0.02**	**<0.0001**
**MYC**	3.25 (69)	3.00 (6)	2.13 (8)	1.75 (69)	0.67	0.13	**<0.0001**
**RASSF1**	5.75 (67)	3.00 (3)	5.00 (6)	2.75 (67)	0.54	0.38	**<0.0001**
**ESR1**	6.25 (68)	5.75 (7)	4.25 (8)	2.75 (68)	0.49	0.29	**<0.0001**

^a^ p-values from non-parametric Mann-Whitney-Wilcoxon test. In bold p-value <0.05, considered statistically significant. T: breast tumor; N: normal breast; L: lymph node; B: blood.

### Cluster analysis

Hierarchical cluster analysis was performed on 64 male breast tumors, for which data on promoter methylation levels of all genes analyzed were available (Figure 1B). Two groups of clustered MBC cases emerged, one group characterized by high (≥30%) *hTERT* promoter methylation levels and a second group characterized by moderate (≥20% and >30%) and low (<20%) *hTERT* promoter methylation levels. Notably, the majority of *BRCA1/2* mutation carriers (8/10) were included in the groups of cases characterized by high and moderate *hTERT* promoter methylation levels (Figure 1B).

### Association between gene methylation and clinical-pathologic characteristics of MBC

For each gene, association between median promoter methylation levels and relevant clinical-pathologic characteristics of each MBC case was evaluated. As shown in Table 3, significantly higher *RASSF1* methylation levels were observed in cases positive for *BRCA1/2* mutation (p=0.008), HER2 expression (p=0.01), and with high tumor grade (G3) (p=0.008). Significantly higher *AR* methylation levels were observed in *BRCA1/2* wild-type cases (p=0.016) and higher *WNT1* methylation levels were observed in PR negative cases (p=0.014).

**Table 3:** Associations between gene methylation levels and clinical-pathologic characteristics in male breast tumors

	hTERT		ESR1		RASSF1		AR		MYC		WNT1	
	%^a^	p^b^	%^a^	p^b^	%^a^	p^b^	%^a^	p^b^	%^a^	p^b^	%^a^	p^b^
**Family history of breast/ovarian cancer**												
Negative	25.5	0.7	6.3	0.7	5.6	0.8	12	0.4	3.3	0.7	6.5	0.2
Positive	32.2		5.8		6		11.7		2.8		7.8	
**Personal history of other cancer**												
Negative	30.2	0.12	6.4	0.2	6	0.5	11.7	0.2	4	0.1	6.5	0.8
Positive	17.7		3.8		4.8		13		3.8		6.8	
***BRCA1/2* status**												
*BRCA1/2* mutated	39.7	0.076	6.8	0.07	9.1	**0.008**	5.2	**0.016**	4.3	0.2	6.8	0.5
*BRCA1/2* wild-type	25.1		5.8		4.7		10.4		3.3		5.9	
**ER**												
Negative	25.0	0.4	6.8	0.3	8.9	0.6	4.6	0.6	3.8	0.5	7.3	0.6
Positive	29.3		5.8		5.6		10		3.3		6.8	
**PR**												
Negative	45.5	0.16	6.6	0.3	5.5	0.16	8.8	0.9	3.5	0.5	8.5	**0.014**
Positive	26.3		5.8		6.5		10.0		3.3		5.5	
**HER2**												
Negative	28.0	0.5	6.2	0.3	4.8	**0.01**	10.0	0.6	3.5	0.08	6.8	0.1
Positive	21.6		4.1		7.4		11.2		2.3		5.1	
**Ki67**												
Negative	25.3	0.6	4.9	0.7	5.5	0.4	10.0	0.6	3.25	0.5	6.8	1
Positive	30.3		6.3		6.0		10.7		3.25		6.9	
**Hystological grade**												
G1+G2	29	0.9	5.8	1	4.8	**0.008**	10.7	0.3	3.4	0.1	6.5	0.2
G3	28.3		6.0	7.8	8.4		2.2		7.9			

^a^%: median of methylation for each gene; ^b^p value derived from Kruskal-Wallis test. In bold p-value <0.05, considered statistically significant.

## DISCUSSION

In order to investigate whether epigenetic signatures could define molecular subgroups of MBCs, we examined methylation profiles of MBC cases, characterized for *BRCA1/2* mutation status, by performing promoter methylation analysis of genes representative of cellular pathways known to be involved in breast cancer. We also analyzed possible correlations between methylation levels of these genes and the main clinical-pathologic characteristics of MBC cases.

Promoter methylation status was assessed by pyrosequencing, a technique that offers a unique opportunity to quantify, site-specifically, the methylated fraction in CpG site. This method has been shown as the most suitable to determinate very low methylation levels and to discriminate between small differences in the methylation status of gene promoters [29].

Methylation analysis was performed in male breast tumors, paired blood samples, normal breast tissue and lymph nodes samples. The analysis results allowed for the identification of tumor specific methylation profiles.

In agreement with previous papers [22–25], our results showed that high methylation levels in promoter regions of candidate genes are frequently observed in male breast tumors. Notably, in our study we were able to obtain and compare data on methylation in male breast tumors and matched blood samples. At present only four studies examined methylation in MBC but none has investigated methylation in DNA from paired blood samples [22–25]. Here we showed that compared with paired blood samples, tumors displayed significant higher methylation levels for all the genes analyzed. Further studies on DNA methylation in blood and normal tissues of MBC patients and population controls should be performed in order to investigate a possible role of methylation as marker for MBC risk and early diagnosis.

In our study, *AR, RASSF1* and *hTERT* were the genes that most frequently showed higher methylation levels in tumor compared to normal breast tissues, with *AR* showing a statistically significant difference. To our knowledge, *AR* and *hTERT* promoter methylation has not been previously investigated in MBC.

We observed high promoter methylation levels of *AR* in the vast majority (94%) of MBC cases examined. AR is known to be involved in a complex network of signaling pathway that collectively regulate cell proliferation [30]. Hypermethylation of *AR* promoter was reported to be associated with reduced AR expression in breast cancer cell lines [31]. Specifically for MBC, AR expression has been reported as a positive prognostic marker for overall and disease-free survival [32]. Notably, AR has also received attention as a valid drug target in MBC patients [33]. Overall, our data add further evidence to a relevant role of AR in MBC and suggest that methylation status of *AR* promoter may eventually impact on the clinical management of MBC patients.

We also observed high promoter methylation level of *hTERT* in a large percentage of male breast tumors (63.8%) and, by cluster analysis, two subgroups of MBC cases were identified based on methylation levels of *hTERT*. *hTERT* encodes for the human telomerase reverse trancriptase (hTERT), the catalytic subunit of telomerase, and plays a key role in telomerase activity [34–35]. The telomerase activity is almost silenced in normal somatic cells, but activated in more than 90% of cancers [36]. Increased expression of the protein associated to hypermethylation of regulatory region of *hTERT* has been reported in cancer cells [37–38]. We showed that a large percentage of male breast tumors are characterized by high promoter methylation level of *hTERT* thus suggesting that telomerase activity may be altered in MBC. Functional studies would be needed to support this hypothesis.

In agreemet with previous studies [23, 25], we showed that *RASSF1* promoter methylation is frequently observed in MBCs. Furthermore, we found associations between *RASSF1* promoter methylation and aggressive tumor characteristics, such as HER2 expression and high tumor grade (G3). The association between *RASSF1* methylation and adverse phenotypic features in MBC may indicate *RASSF1* as a prognostic biomarker in MBC.

*ESR1* promoter methylation has been reported as an independent biomarker for aggressive MBC, due to its correlation with high mitotic count and high tumor grade [22]. In our MBC series no significant correlation between *ESR1* promoter methylation status and clinical-pathologic characteristics of tumors emerged, however higher *ESR1* methylation levels were observed in cases with biological variable indicative of a more aggressive phenotype, such as ER-, PR-, G3, Ki67/MIB1+.

Although knowledge on methylation profiles of MBC is increasing, specific comprehension on methylation profiles of MBCs associated with *BRCA1/2* mutation status is still incomplete. In our MBC series, statistically significant differences in gene-specific methylation profiles related to *BRCA1/2* mutation status were observed. In particular, according to Deb et al 2017 [25], significantly higher promoter methylation levels of *RASSF1* were associated with *BRCA1/2* positive status. In addition, we showed that higher *AR* methylation levels were found in *BRCA1/2* wild-type cases. These findings add new molecular evidence on the distinction between sporadic and hereditary MBC.

In summary, our results indicate that alterations in gene methylation profiles are common in MBC and that tumor-associated gene methylation patterns may identify specific MBC subgroups related to *BRCA1/2* mutation status and clinical-pathologic characteristics. Overall these findings may allow for the identification of molecular predictive and prognostic biomarkers and may have implications for clinical management of MBC patients. Further studies, particularly on series of MBCs with adequate follow-up, are needed in order to support the clinical relevance of gene promoter methylation as potential molecular biomarkers.

## MATERIALS AND METHODS

### Patient samples

DNA from tumor and paired blood samples of 69 MBCs, 8 normal lymph nodes and 7 normal male breast tissues was analyzed. All cases were recruited from a population-based series of MBC and were characterized for *BRCA1/BRCA2* mutations and for main clinical-pathologic features including: family and personal history of cancer, ER, PR, HER2 and Ki67/MIB1 expression, and tumor grade (G).

The entire *BRCA1* and *BRCA2* coding sequences were analyzed mostly by automated Sanger sequencing, otherwise by a combination of protein truncation test (PTT) and single-strand conformation polymorphism (SSCP). All cases were retested for *BRCA1/2* mutations using Next Generation Sequencing. Cases were also tested by MLPA for the detection of large genomic rearrangements [26, 39–40].

The expression of ER, PR, and Ki67/MIB1 was scored based on the percentages of positive nuclei (ER/PR positive if >10%; Ki67 high if >20%) over the total number of counted cancer cell nuclei. HER2 expression was scored according to the percentage of positive tumor cells as: 1+ (<25%), 2+(25-50%), 3+ (>50-75%) and 4+ (>75%). HER2 positivity was defined as a score of 2+ using immunohistochemistry (IHC) test, or amplification shown by fluorescence *in situ* hybridization (FISH), in equivocal cases [41–42].

For some cases the amount of DNA was inadequate to carry out all molecular analyses.

The participants signed an informed consent form with a detailed description of the study protocol. The study was approved by The Local Ethical Committee (Sapienza University of Rome, Prot 669/17).

### DNA extraction and methylation analysis

Genomic DNA was isolated from blood using ReliaPrep^TM^ Blood gDNA MiniPrep System (Promega) according to the manufacturer's instructions. DNA from tumor, normal breast and lymph node samples was extracted from formalin fixed paraffin-embedded (FFPE), using 5-10um thick sections, by EpiTect Plus FFPE lysis kit (Qiagen) according to the manufacturer's instructions. Tumor DNA was extracted from microdissected tumor samples. Microdissection assures that each sample contains at least 60-70% of tumor cells.

DNA bisulfite modification was performed using EpiTect Plus DNA Bisulfite kit (Qiagen) according to the manufacturer's instructions.

Promoter methylation of *AR, ESR1, hTERT*, *MYC*, *RASSF1* and *WNT1* genes for a total of 26 CpG sites, was evaluated.

Methylation analysis was performed by pyrosequencing, a highly sensitive and reproducible method, which provides absolute quantitative information on bases at each CpG site analyzed, using Pyromark 24Q (Qiagen) platform. Pyrosequencing for DNA methylation analysis was performed following protocol previously described [29].

Specific pyrosequencing primers were used to assay on consecutive series of 3 to 5 CpG sites in the promoter region of the selected genes. For *hTERT* gene, primer for PCR amplification and sequencing were designed using the PyroMark Assay Design 2.0 software. Primers were designed to amplify fragments of about 90bp because of possible fragmentation of DNA isolated from FFPE samples. For the other five genes commercially available assays, including primers for amplification and sequencing, were used (Qiagen).

The degree of methylation at each CpG position in a sequence was determined from the C/T ratio. Target CpGs are evaluated by converting the resulting pyrograms to numerical values for peak heights. For each analyzed methylation levels were expressed as median of methylation percentage at all CpG sites gene both in tumors and normal tissues.

### Statistical analysis

The non-parametric Mann-Whitney-Wilcoxon test was used to compare DNA methylation values in normal (blood, lymph node and breast tissue) and tumor samples.

Unsupervised hierarchical clustering was performed to analyze relevant clusters and co-methylation. Dendrograms and heatmap were then generated using Euclidean distance matrix and complete linkage.

To assess associations between methylation levels for each gene and clinical-pathologic features in the tumor sample group, the Kruskal–Wallis test was used.

For all the analyses, a p-value <0.05 was considered statistically significant. All statistical analyses were performed with the R software (www.r-project.org).

## Abbreviations

MBC: Male breast cancer
FBC: female breast cancer
G: tumor grade
ER: estrogen receptor
PR: progesterone receptor
